# Concomitant activation of D_1_ dopamine and α_2A_ adrenergic receptors improves cognition compared with methylphenidate

**DOI:** 10.21203/rs.3.rs-7861542/v1

**Published:** 2026-01-22

**Authors:** Luke Bransom, Ava P. Bassett, Mi Zhou, Richard B. Mailman, Yang Yang

**Affiliations:** 1 Department of Neuroscience and Experimental Therapeutics, Penn State University College of Medicine, Hershey PA 17033, United State; 2 Department of Neurology, Penn State Milton S. Hershey Medical Center, Hershey PA 17033, United State

**Keywords:** working memory, cognitive flexibility, D_1_ dopaminergic receptor agonist, α_2_ adrenergic receptor agonist, methylphenidate

## Abstract

**Rationale:**

Methylphenidate is commonly prescribed to manage symptoms of attention-deficit/hyperactivity disorder (ADHD), but like other stimulants it has limited effectiveness. Methylphenidate works by increasing the synaptic availability of dopamine and norepinephrine, resulting in stimulation of dopaminergic and adrenergic receptors. One hypothesis is that selective receptor targeting may be more effective clinically and have fewer side effects than non-selective stimulants.

**Objectives and Methods:**

To test this hypothesis, we compared methylphenidate with three compounds: the selective D_1/5_ dopamine agonist 2-methyldihydrexidine; the selective α_2A_ adrenergic agonist guanfacine; and the cannabinoid compound cannabigerol that has α_2A_ agonist properties. Acute effects on temporal order memory, cognitive flexibility, and spatial working memory were evaluated using two rodent behavioral tasks.

**Results:**

Co-administration of an α_2A_ agonist and a D_1_ agonist produced greater cognitive improvement than methylphenidate. The performance improvement from these selective agents, however, was only observed in rats that had poor performance at baseline.

**Conclusions:**

These findings suggest that synergistic effects may emerge from the coadministration of selective agents (e.g., α_2A_ and D_1_ agonists) and should be considered for further study, especially as regards individuals with decrements in cognitive function.

## Introduction

Attention-deficit/hyperactivity disorder (ADHD) is one of the most common neurodevelopmental disorders, affecting about 9% of children, that often persists throughout adulthood [1, 2]. This cognitive disorder is categorized by impulsivity and inattention that negatively impacts learning and social behavior [3, 4]. Besides the well-known symptoms of ADHD, such as reduced working memory (WM), struggling to make decisions, and difficulties sustaining attention, individuals who have this disorder tend to have higher instances of substance abuse, anxiety, and depression [2, 5, 6]. Most importantly, there are no known cures for ADHD, and patients are generally required to take medication (e.g., stimulants) indefinitely.

ADHD has a complex and heterogeneous etiology involving many unknown factors (genetic, developmental, environmental); this complicates a search for personalized therapy [7, 8]. Thus, the gold standard of care for ADHD has been formulations of stimulants such as methylphenidate (MPH) and amphetamine [9–11]. The exact mechanisms of how MPH and other stimulants affect ADHD are not fully understood [12], but MPH affects numerous receptor systems via the inhibition of catecholamine reuptake (i.e., of dopamine and norepinephrine). This explains why these compounds have undesired side-effects or risk of abuse. Although the complex etiology of ADHD may make precision medicine approaches difficult, improved symptomatic control is still very important to a majority of patients.

MPH is an indirect agonist of adrenergic and dopaminergic receptors like the α_2A_ adreno receptor (α_2A_R) and the D_1_ dopamine receptor (D_1_R) [13–17]. Activation of the α_2A_R is known to enhance prefrontal cortical (PFC) function and network connectivity [18], and dysfunction of this system is common in ADHD [14]. The D_1_R also plays an important role in optimizing PFC activity, and aberrant dopamine signaling in the PFC leads to deficits in WM and other cognitive processes [19–21], which are also seen with dysfunctional norepinephrine signaling [22]. Thus, both the α_2A_R and D_1_R systems play pivotal roles in promoting optimal PFC function; the α_2A_R enhances network connectivity, whereas the D_1_R finely tunes the signal-to-noise ratio by regulating excitatory and inhibitory balance [18, 23–28]. Increasing either dopamine and norepinephrine signaling results in an inverted-U-shaped dose-response curve; too little or too much causes suboptimal cognitive processes [27, 29–34].

It has been speculated that individuals with ADHD have dysregulated signaling in one or both of these systems [25], suggesting them to be useful therapeutic targets. In fact, guanfacine (GFX), a selective α_2A_R full agonist, is approved for clinical use in the treatment of ADHD and is a useful alternative option to stimulants [13, 35–38]. It is, however, noteworthy that a recent study indicated GFX slightly improved WM, though not as effectively as MPH [36]. On the other hand, our lab, as well as others, have reported that selective D_1_ agonists improve both spatial WM and temporal order memory in rodents, with the effects in our study surpassing those of MPH [29–33, 39]. Based on this collective evidence, we therefore sought to test the hypothesis that D_1_ and α_2A_ coactivation might work additively or synergistically in improving cognitive processes, and thus compared them alone or in combination versus the standard-of-care methylphenidate. We used two well-established behavioral paradigms: temporal-order object recognition (TOR) in the open field and delayed alternation response (DAR) in the T-maze. The TOR task measures object recognition with a focus on the temporal order, while the DAR task measures spatial WM. Both tasks critically rely on PFC function, as lesioning the PFC hindered rodents’ ability to perform these two tasks [39, 40]. More importantly, patients with ADHD experience a reduction in both temporal order memory and spatial WM [41, 42]. We hypothesized that a selective α_2A_ agonist and a selective D_1_ agonist, whether administered alone or together, will more effectively rescue these memory deficits than MPH. Furthermore, we hypothesize that cotreatment of an α_2A_ agonist and a D_1_ agonist will be more efficacious than either compound alone.

## Materials and Methods

### Subjects

A total of 31 male Fischer rats were included in the study, with 24 obtained from Envigo (Frederick, MD) and seven from the National Institute on Aging (Hollister, CA; Raleigh, NC; or Kingston, NY). At the start of the experiments, rats weighed between 295 and 495 g. Depending on their body weight, they were housed individually, in pairs, or in groups of three, under a 12-hour light/dark cycle. Water was available at all times. Animals assigned to the TOR task were given free access to food, whereas those in the DAR task were maintained on a restricted diet of Bio-Serv rat chow to keep their body weight at 90–95% of their free-feeding weight, allowing food to be used for motivation. A palatable chocolate-flavored sucrose pellet (Bio-Serv, Flemington, NJ) was used as the behavioral reward. All animal care and experimental procedures were conducted in accordance with the National Institutes of Health Guide for the Care and Use of Laboratory Animals and Penn State Hershey Animal Resources Program, with approval from the Institutional Animal Care and Use Committee (IACUC) of Penn State College of Medicine.

### Drug preparation and administration

All drug solutions were freshly prepared on the day of experimentation. MPH (methylphenidate, Ritalin^®^) powder was gifted by Novartis. It was dissolved in purified water (10 mg/mL) and then mixed into Nutella^®^ to produce an oral dose of 1.5 mg/kg, corresponding to the allometric equivalent used in ADHD patients [17, 43, 44]. Nutella alone served as the vehicle control. The D_1_ agonist 2-methyldihydrexidine (2MDHX) was synthesized following a modified protocol [45, 46]. Because 2MDHX are prone to oxidation, it was prepared in 0.1% ascorbic acid vehicle and administered subcutaneously due to its limited oral bioavailability. A dose of 3 g/kg was selected based on prior studies demonstrating its efficacy in enhancing WM in rats [29–32]. The α_2A_ agonist GFX (guanfacine, Intuniv ER^®^) was purchased from Tocris (Bristol, UK). It was dissolved in saline vehicle and administered intraperitoneally at a 0.1 mg/kg dose. This dose was chosen because it improved WM in rats without causing hypotension [47, 48]. Cannabigerol (CBG) is a cannabinoid that has α_2A_ agonist activity, but is very low CB1 or CB2 receptor activity [49, 50]. Given the increased recreational use of cannabis, including among individuals with ADHD [51], we decided to include CBG in this study. It was purchased from Cayman Chemical (Ann Arbor, MI) and dissolved in saline based vehicle (1:1:18 DMSO:Tween80:Saline) for intraperitoneal administration at 1 mg/kg. This dose was chosen because it was reported to be functionally equivalent to GFX in terms of cardiovascular effects, but has a lower risk of hypotension in mice [52].

### Temporal-order Object Recognition (TOR) test

#### Apparatus

Open field observational chambers (E63–20, Coulbourn Instruments, Whitehall, PA) were used for the temporal-order object recognition (TOR) experiments. The chambers were 41 × 41 cm squared, and made of opaque walls, with black floors. CCD cameras (30 frames/second, STC-TB33USB-AS, SenTech, Carrollton, TX) were set above to track the free movement in real time, and footage was analyzed in LimeLight software (Actimetrics, Coulbourn Instruments, Whitehall, PA). Several types of objects were used, including opaque plastic bottles and enrichment toys from Bio-Serv (Kong Genius, Kong Toys, Precious Gem, Dumbbells; Bio-Serv, Flemington, NJ). They all were as tall as or no taller than twice the size of the rat [53, 54].

#### Behavioral task procedure

**Fig. 1:**
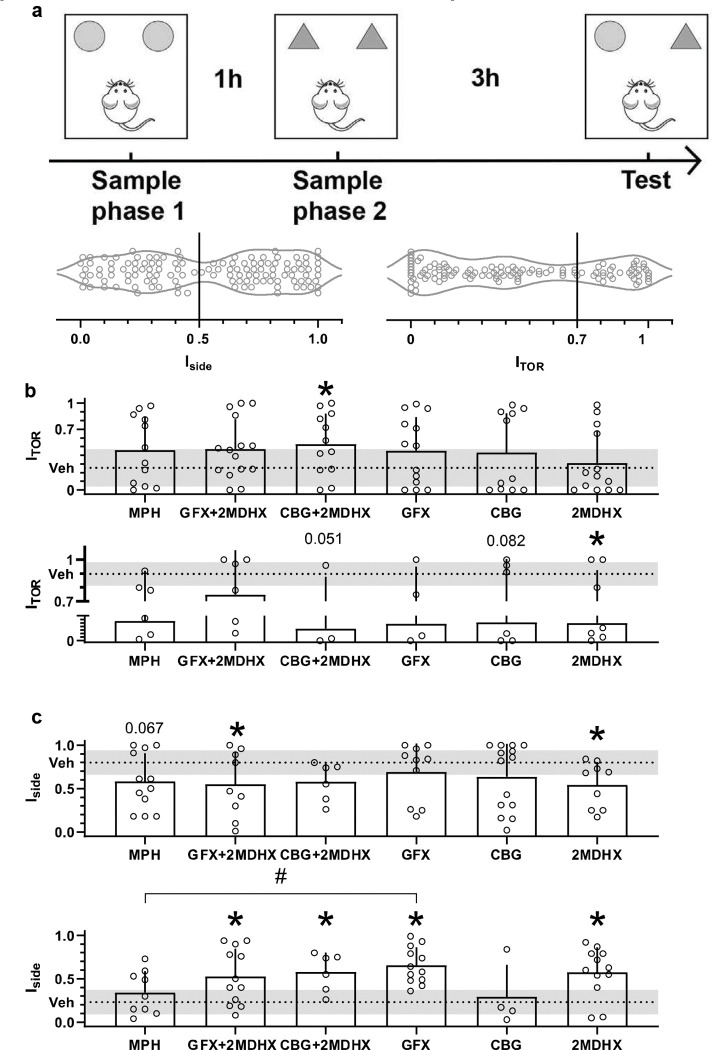
Comparison of treatment effects on TOR task performance. **a**: Schematic of TOR task and rats’ baseline performances when vehicle was administered. Each circle represents an individual rat. Note the large variation among rats. I_TOR_=0.7 was used as the criteria to categorize rats into groups having inferior (≤0.7) or superior (>0.7) baseline WM; I_side_=0.5 was used as the criteria to categorize rats into flexible (<0.5) or rigid (≥0.5) performance groups. **b-c**: Changes of I_TOR_ (**b**) and I_side_ (**c**) under various drug conditions. Top row from each panel shows the results from rats whose baseline performances showed poor WM-related temporal-order object recognition (**b**) or cognitive flexibility (**c**); bottom is for the rats having better temporal-order recognition (**b**) or cognitive flexibility (**c**). Bars represent mean ± SD, and gray shades represent means at the baseline when vehicles were administered. * indicates significant difference from vehicle, and # indicates significant difference between two drug conditions. Values above bars are p values nearing statistical significance.

Rats were habituated to all procedures and tested in a modified classic novel object recognition test (Figure 1a). There was first a three-minute habituation phase. After habituation, rats performed the acquisition phase that consisted of two sampling trials, followed by the retrieval phase that consisted of one choice trial [55–57]. Each trial lasted five minutes, and rats were returned to their home cages after each of these five-minute explorations in the test chambers. For the first sampling trial, each rat was presented with two identical copies of one object (O1+O1). The second sampling trial, one hour after the first, was similar except the rat would be presented with two identical copies of a second object (O2+O2) located where the O1s were during the first sampling trial. After a three-hour delay, the choice trial involved presenting a third copy for each of the two prior objects (O1+O2). The arena was cleaned with 70% EtOH between each trial. The objects were randomly assigned for each test session, as well as the location that two objects were assigned during the choice trial (i.e., O1 at left vs O2 at left).

#### Experimental design

A total of 22 rats were used for this behavioral paradigm, and were habituated to all procedures before the start of the experiments. To ensure that the observed behavioral changes were induced by drugs, there was always a vehicle test day right before a drug test day to serve as the reference. The vehicle test day used 0.1% ascorbic acid (sc), saline (ip), Nutella (pos), or their combination. The drug test day had one of the first four drug combinations (Table 1) administered. The drug or vehicle administration was right after the three-minute habituation phase and 15 minutes before the first sampling trial of the TOR test. After a drug test day, there were at least five consecutive days in which no substance was given for “washout” prior to reusing the animal. The test order of the first four drug combinations was randomized for each rat. Once these four drug combinations were tested for a rat, another two drug combinations (i.e., CBG replacing GFX) were tested in a counter balanced order in all rats (Table 1). The entirety of this experiment was completed in approximately four months.

### Delayed alternation response (DAR) test

#### Apparatus

A standard T-maze was used, which had one start runway (56 cm long, 10 cm wide, 18 cm high) and two finish arms (41 cm long, 10 cm wide, 18 cm high). The lower portion of the start arm served as the start box, which could be cordoned off by a solid gate. At the intersection of the maze, the runways to the two finish arms, left or right, also can be cordoned by solid gates. A CCD camera (30 frames/second, STC-TB33USB-AS, SenTech, Carrollton, TX) was mounted directly above the maze to capture animal movement. Video data were collected using the Limelight video recording system (Actimetrics, Coulbourn Instruments, Whitehall, PA). Pre-defined zones and grids were used to quantify behavioral parameters such as decision latency (time spent in the choice zone) and arm selection (grid crossings). The Limelight software automatically computed the duration spent in a zone and recorded the time point at which the animal crossed a grid.

#### Behavioral task procedure

**Fig. 2:**
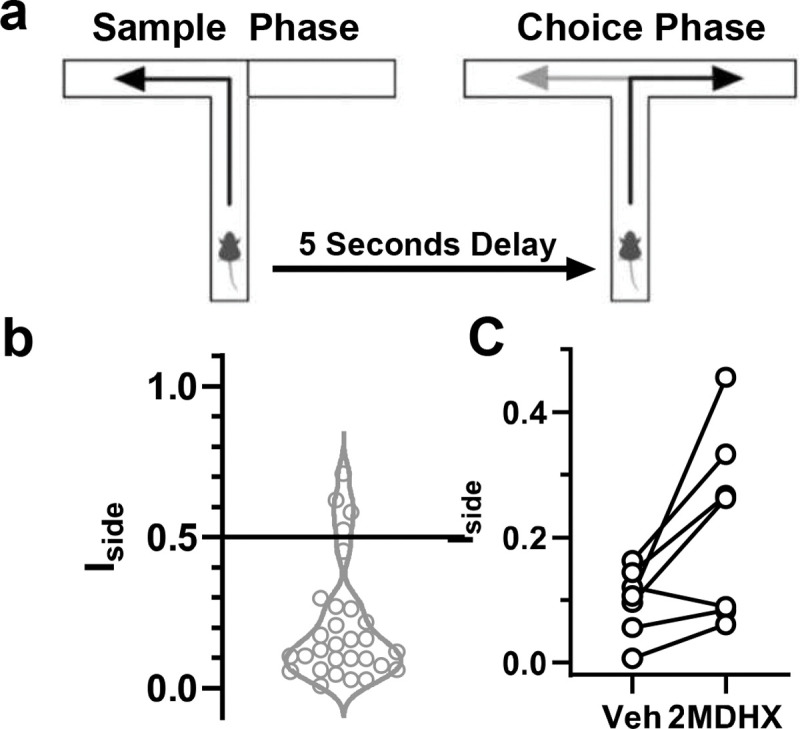
Comparison of treatment effects on DAR task performance. **a:** Schematic of DAR task in the T-maze. Note that the sample run cordons off one of the choice arms, while the choice run has both arms accessible. Black arrow indicates correct choice; gray arrow represents incorrect choice. **b:** Behavioral index used to quantify cognitive flexibility (I_side_). Note that the majority of rats showed no side bias (I_side_<0.5) at the baseline when vehicles were administered. **c:** Changes of I_side_ under 2MDHX treatment compared to its vehicle. Each line connects the data from the same individual rat. Data from the only one rat having rigid side bias (I_side_≥0.5) was not included.

This is a discrete T-maze alternation task where each trial was composed of two runs (Figure 2a). Each run began when the rat was released from the start box after the tester raised the gate. The first run was the sample phase in which the runway to one of the two (left or right) finish arms was randomly cordoned by a solid gate. The rat would enter one of the finish arms during this sample phase, and then gently picked up and placed back in the start box for a predetermined five seconds delay [a general temporal scale of WM tasks [58]]. After the delay time the rat was released from the start box again, and the second run, the choice phase, began. During the choice phase, neither left or right finish arms was cordoned, and the rat was free to choose either finish arm. There was no visual, odor, or audio cue for the choice. The rat intrinsically tends to explore novel places and therefore should visit the arm that was cordoned in the first run and thereafter not explored during the sample phase. This was reinforced with a hand-fed food reward after the rat had made the turn. On the contrary, the wrong/incorrect choice (i.e., the rat continuously visited the same arm that was not cordoned and thereafter had been explored during the sample phase) led to no reward. The completion of the choice phase was when the rat was gently picked up from one of the finish arms and placed back in the start box. The second trial then began. This was repeated for 10 trials for one test session to be completed. For each run during each trial, rats needed to run from the start box to the intersection and then turn to one of the finish arms in less than two minutes, otherwise the trial was aborted, and the rat was gently picked up and placed back in the start box to restart. The maze was cleaned with 70% EtOH between runs and trials.

#### Experimental design

A total of nine rats were used. They were trained once per day and given rest on weekends. To ensure acclimation to experimental handling, they underwent mock sessions in which all procedures were simulated without actual drug administration (e.g., needle insertion without injection). Once they were well trained, the test session was implemented on the next day. First was the vehicle session where 0.1% ascorbic acid (sc), saline (ip), Nutella (os), or their combination was administered; the drug session was then implemented on the next day where one of the first four drug combinations (Table 1) was administered. The test order of these four drug combinations was randomized for each rat. CBG was not evaluated in this behavioral test because of prolonged training. All drug and vehicle treatments were administered 20 minutes prior to the task. Following each drug testing session, a minimum five-day “washout” period was implemented before the same animal was tested again. During this interval, rats continued their routine behavioral training sessions without receiving any drug or vehicle treatments. This experiment was finished for each rat in about 1–3 months.

### Data analysis

All analyses were done using SPSS 30 and GraphPad Prism 10. Data are presented as mean ± standard error (SE) if not mentioned otherwise. Normality was checked by Kolmogorov-Smirnov test and Shapiro-Wilk test. Both the Linear Mixed Model and the paired t-test were used to determine whether behavioral performance differed across drug conditions or from the vehicle. A p-value of < 0.05 was considered statistically significant, with multiple comparison corrected by Bonferroni.

## Results

### Effects on TOR task

TOR task performance relies on multiple cognitive processes. Among them, WM-related temporal order object recognition was quantified by the percentage of time a rat explores the novel object (O1) during the choice trial in the retrieval phase [i.e., index ***I***_***TOR***_**=*O1/(O1*+*O2)***, where O1 and O2 represent the time a rat interacts with the object O1 or O2, respectively [33]]. Overall, there was no significant difference when comparing I_TOR_ amongst different drug conditions. It is noteworthy, however, that the spread of I_TOR_ under vehicle condition was wide, ranging from 0 to 1, with coefficient of variation as high as 78% (Figure 1a).

Lower I_TOR_ is interpreted as representing poor WM. We then sought to determine if our compounds effectively rescued poor WM on rats who had lower I_TOR_. Based on the vehicle session, rats were split into either a “superior” (I_TOR_>0.7) or “inferior” (I_TOR_≤0.7) performing group [33]. 2MDHX cotreatment with CBG significantly increased I_TOR_ of rats with poor baseline WM (Figure 1b; 2MDHX+CBG vs vehicle = 0.526±0.103 vs 0.256±0.024; p=0.048, t147=3.107). Interestingly, despite the rescue effect from 2MDHX and CBG coadministration, neither compound significantly increased I_TOR_ when administered alone (Table 2). On the other hand, for rats with superior baseline WM, 2MDHX alone significantly decreased I_TOR_ (Figure 1b; 2MDHX vs vehicle = 0.496±0.162 vs 0.898±0.014; p=0.035, t58=3.298); all other compounds caused only trends (Table 3).

Besides temporal order recognition, rats exhibited preference to explore objects located on either the left or right side during two sampling trials when WM was less needed. This side bias was quantified by the index ***I_side_*** = |(***O_l_*** − ***O_r_***)/(***O_l_*** + ***O_r_***)|, where OL and OR represent the average time a rat interacts with the object located on its left or right side, respectively. High I_side_ indicates strong side bias, implying less cognitive flexibility, and was observed on many rats in this study (Figure 1a). We then examined if our compounds effectively improved the flexibility on rats who had higher I_side_. Based on the vehicle session, rats were split into either a “flexible” (I_side_ <0.5) or “rigid” (I_side_ ≥0.5) performing groups. 2MHDX alone, as well as its coadministration with GFX, significantly decreased I_side_ of rats who had rigid baseline performance (Figure 1c; 2MDHX vs vehicle = 0.541±0.088 vs 0.800±0.017, p=0.036, t127=3.206; 2MDHX+GFX = 0.549±0.125, p=0.045, t127=3.117). Similarly, all the other drug conditions showed only trends (Table 4). Interestingly, for rats with flexible baseline performance, MPH and CBG did not change their I_side_ (Figure 1c; Table 5), whereas other drug conditions, i.e., 2MDHX (2MDHX vs vehicle = 0.577±0.082 vs 0.231±0.018; p=0.006, t106=3.719), GFX (GFX = 0.654±0.060; p<0.0001, t106=7.418), and cotreatment of 2MDHX with GFX (2MDHX+GFX = 0.528±0.093; p=0.024, t106=3.275) or CBG (2MDHX+CBG = 0.580±0.091; p=0.009, t106=3.592), all significantly increased I_side_. The I_side_ after GFX administration was significantly different from the I_side_ under MPH treatment (MPH = 0.342±0.083; p=0.011, t106=3.534).

### Effects on DAR test

Spatial WM-related T-maze alternation was quantified by the percentage of alternated choices a rat made over all completed trials. Overall, the alternation rate was not significantly changed under any drug condition (Table 6). Besides alternation, rats exhibited preference to explore the left or right arm during the sample phase when rats can only explore one arm without choice and WM was not needed. This side bias was quantified, similarly as for the TOR task, by the index ***I_side_*** = |(***T_l_*** − ***T_r_***)/(***T_l_*** + ***T_r_***)|, where TL and TR represent the average time a rat takes to turn into the left or right arm in the T-maze, respectively. Only one rat showed strong side bias (I_side_ ≥0.5) in the T-maze, whereas others were relatively flexible (I_side_ <0.5; Figure 2b). We then examined if our compounds impaired rats’ cognitive flexibility. Only 2MDHX alone, when compared with its vehicle, tended to increase I_side_ (Figure 2c; 2MDHX vs vehicle = 0.221±0.056 vs 0.100±0.020; p=0.040 before Bonferroni correction, t6=2.604). All other drug conditions did not change I_side_ (overall Linear Mixed Model result: p=0.409, F4,45=1.016; p>0.05 for all pairwise comparisons).

## Discussion

This study sought to determine whether co-administration of an α_2A_ agonist and a D_1_ agonist would evoke synergistic or additive effects on rescuing deficits in spatial WM and/or temporal order memory in rats. In addition, we also investigated if selective α_2A_ agonists and D_1_ agonists, either administered alone or together, would be more effective than the non-selective stimulant, MPH.

We found that when the selective full D_1_ agonist 2MDHX was administered concomitantly with a selective α_2A_ agonist (either CBG or GFX), either temporal order memory or cognitive flexibility was improved in rats with deficits. MPH, on the other hand, did not improve temporal order memory, spatial WM, or cognitive flexibility. A trend toward lowering rigidity in cognitive flexibility was observed with MPH, but the effect was not statistically significant. These findings are in general agreement with existing literature that suggests that the effects of MPH on memory and cognitive functions could be task-dependent [59–62]. Our previous study [32] reported similar results, showing that MPH improved spatial WM in some rats performing a continuous T-maze alternation task, but impairing performance in others. The current findings underscore the limitations of using stimulants, such as MPH, to treat ADHD, mirroring observations in patients [63, 64]. While it is reasonable to suggest using a behavioral paradigm where MPH has shown more consistent efficacy, our objective was to use tasks that probe specific cognitive domains often impaired in ADHD but less reliably improved by MPH. This approach allowed us to evaluate whether selective α_2A_ agonists and D_1_ agonists can better address these particular cognitive domains. Our data supports the notion that cotreatment of selective agents, such as an α_2A_ agonist with a D_1_ agonist, could be an alternate, potentially superior therapeutic option. Future studies will extend this work across a broader range of behavioral paradigms to further characterize the relative strengths and limitations of these treatment strategies.

The benefits of co-treatment, however, were tempered by the detrimental effects on rats that were performing well already. Co-administration of the selective D_1_ agonist 2MDHX with the selective α_2A_ agonist CBG or GFX, not only significantly lowered cognitive flexibility, but also tended to impair temporal order memory. Similar detrimental effects were observed with monotherapies as well. When administered alone, 2MDHX significantly impaired both cognitive flexibility and temporal order memory. When either GFX or CBG was administered alone, there was significantly decreased cognitive flexibility or a trend to impair temporal order memory. One limitation of these studies was that the drug dose chosen might have been on the descending phase of the inverted-U dose response curves of these rats with superior baseline performance. Additionally, although the compounds used are receptor-selective, off-target interactions cannot be entirely ruled out and could potentially lead to adverse effects. However, given the low dose administered and the fact that these adverse effects were observed only in the good-performing group, off-target effects are unlikely to be the primary mechanism. It is interesting to note, however, that when compared to these selective agents, the non-selective drug, MPH, was the only treatment that did not negatively affect cognitive flexibility or temporal order memory in rats with superior baseline performance. These findings support the hypothesis that when brain activities, such as those in the PFC, are operating normally, they are more susceptible to disruption by selective agents, such as D_1_ agonists and α_2A_ agonists. The greater risk of perturbation is likely to lead to a more significant imbalance in signaling, ultimately impairing cognitive functions. This hypothesis merits further investigation.

Our results also suggest that D_1_Rs may play a more critical role than α_2A_Rs in regulating both cognitive flexibility and temporal order memory. The selective full D_1_ agonist 2MDHX alone was capable of lowering rigidity in cognitive flexibility, whereas neither of the α_2A_ agonists GFX or CBG demonstrated a similar benefit. On the other hand, for the rats who had already performed well, 2MDHX alone was capable of causing impairments on both cognitive flexibility and temporal order memory, whereas the detrimental effects of the α_2A_ agonists were more mixed. CBG alone did not affect cognitive flexibility but tended to impair temporal order memory, whereas GFX alone significantly reduced cognitive flexibility without impairing temporal order memory. One resulting hypothesis is that α_2A_Rs require optimal D_1_Rs function, that is, co-administration of a D_1_ agonist with an α_2A_ agonist improved poor temporal order memory, whereas neither an α_2A_ agonist alone, nor a D_1_ agonist alone, produced the same effects. The exact mechanisms underlying these findings require further investigation. One potential explanation is that D_1_Rs function to balance the excitatory-inhibitory neural activities in the PFC, thereby fine-tuning the signal-to-noise ratio to achieve this synergistic effect with α_2A_Rs [23, 34].

Our results also revealed differences between CBG and GFX. CBG had more profound effects on temporal order memory. Its concomitant administration with a D_1_ agonist significantly rescued poor temporal memory, but also tended to cause impairment in rats who had already performed well. These effects were not observed with GFX. Instead, GFX was more effective in modulating cognitive flexibility. Administration of GFX, either alone or in combination with a D_1_ agonist, either significantly improved cognitive flexibility in rats with rigidity or caused impairment in those rats who already had good cognitive flexibility. CBG, by contrast, did not produce the same effects, nor were they as pronounced as those observed with GFX. Both CBG and GFX have α_2A_ agonist activity [49, 50, 65], but CBG also is a 5HT_1A_ antagonist [50]. Indeed, its pharmacological properties of CBG are still not completely defined, and actions at other receptors mechanisms may help explain the different effects observed of CBG and GFX.

## Conclusions

In summary, we found that coadministration of selective agents that are agonists at different receptors, in this case the α_2A_ adrenoreceptor and the D_1_ dopamine receptor, may produce synergistic effects on cognitive rescue. Compared to non-selective stimulants, this approach could offer a promising, potentially superior therapeutic option for ADHD. One caution, however, is that selective agents can pose a greater risk of disrupting brain activities, potentially causing cognitive impairment in subjects who are already well-functioning. Before this idea is translated into human trials, a significant amount of additional research is needed. The complex nature of these experiments made it difficult to do the desired full dose-response studies. Thus, some of the undesired effects on the measured behaviors actually might result from too much synergism between the relevant circuits. In addition, the only D_1_ agonist on the clinical horizon is tavapadon, a partial agonist with significant functional selectivity (i.e., highly biased toward cAMP signaling vs. β-arrestin) that will likely be approved for Parkinson’s disease in the near future [66, 67]. The unique pharmacological profile of tavapadon raises another important variable to study even from the standpoint of acute testing. This arena appears to be a ripe area for future investigation with a number of interesting pharmacological hypotheses that are worthy of testing.

## Figures and Tables

**Table 1:** Drug combinations evaluated by behavioral tests.

		MPH (1.5 mg/kg, po)	2MDHX (3 μg/kg, sc)	GFX (0.1 mg/kg, ip)	CBG (1 mg/kg, ip)
**Drug combinations**	**1**	+	−	−	−
	**2**	−	+	+	−
	**3**	−	+	−	−
	**4**	−	−	+	−
	**5**	−	+	−	+
	**6**	−	−	−	+

Once the first four combinations were tested, the last two were performed (this based on primary and secondary hypotheses). The test order for the first four was randomized. The test order for the last two also was randomized in a counter balanced order among all rats.

**Table 2:** Comparison of treatment effects on rats with lower WM-related TOR task performance (i.e., low I_TOR_) at the baseline.

		p-values					
	mean ± SEM	MPH	GFX + 2MDHX	CBG + 2MDHX	GFX	CBG	2MDHX
**MPH**	0.458 ± 0.112	╲	1.000	**1.000**	1.000	1.000	1.000
**GFX+2MDHX**	0.471 ± 0.089	1.000	╲	1.000	1.000	1.000	1.000
**CBG+2MDHX**	0.526 ± 0.103	1.000	1.000	╲	1.000	1.000	0.720
**GFX**	0.452 ± 0.108	1.000	1.000	1.000	╲	1.000	1.000
**CBG**	0.429 ± 0.137	1.000	1.000	1.000	1.000	╲	1.000
**2MDHX**	0.311 ± 0.103	1.000	1.000	0.720	1.000	1.000	╲
**Vehicle**	0.256 ± 0.024	0.529	0.529	**0.048**	0.234	0.720	1.000

Overall Linear Mixed Model result: p=0.004, F6,147=3.379. p values of pairwise comparisons are reported in the columns next the mean ± SEM.

**Table 3:** Comparison of treatment effects on rats with higher WM-related TOR task performance (i.e., high I_TOR_) at the baseline.

		p-values					
	mean ± SEM	MPH	GFX + 2MDHX	CBG + 2MDHX	GFX	CBG	2MDHX
**MPH**	0.557 ± 0.149	╲	1.000	1.000	1.000	1.000	1.000
**GFX+2MDHX**	0.750 ± 0.130	1.000	╲	0.842	1.000	1.000	1.000
**CBG+2MDHX**	0.340 ± 0.310	1.000	0.842	╲	1.000	1.000	1.000
**GFX**	0.473 ± 0.240	1.000	1.000	1.000	╲	1.000	1.000
**CBG**	0.512 ± 0.202	1.000	1.000	1.000	1.000	╲	1.000
**2MDHX**	0.496 ± 0.162	1.000	1.000	1.000	1.000	1.000	╲
**Vehicle**	0.898 ± 0.014	0.189	1.000	**0.051**	0.146	**0.082**	**0.035**

Overall Linear Mixed Model result: p<0.001, F_6,58_=4.851. p values of pairwise comparisons are reported in the columns next the mean ± SEM.

**Table 4:** Comparison of treatment effects on rats with lower cognitive flexibility (i.e., high I_side_) at the baseline during the TOR task performance.

		p-values					
	mean ± SEM	MPH	GFX + 2MDHX	CBG + 2MDHX	GFX	CBG	2MDHX
**MPH**	0.581 ± 0.094	╲	1.000	1.000	1.000	1.000	1.000
**GFX+2MDHX**	0.549 ± 0.125	1.000	╲	1.000	1.000	1.000	1.000
**CBG+2MDHX**	0.688 ± 0.082	1.000	1.000	╲	1.000	1.000	1.000
**GFX**	0.691 ± 0.104	1.000	1.000	1.000	╲	1.000	1.000
**CBG**	0.636 ± 0.101	1.000	1.000	1.000	1.000	╲	1.000
**2MDHX**	0.541 ± 0.088	1.000	1.000	1.000	1.000	1.000	╲
**Vehicle**	0.800 ± 0.017	**0.067**	**0.045**	1.000	1.000	0.452	**0.036**

Overall Linear Mixed Model result: p<0.001, F_6,127_=4.255. p values of pairwise comparisons are reported in the columns next the mean ± SEM.

**Table 5:** Comparison of treatment effects on rats with higher cognitive flexibility (i.e., low I_side_) at the baseline during the TOR task performance.

		p-values					
	mean ± SEM	MPH	GFX + 2MDHX	CBG + 2MDHX	GFX	CBG	2MDHX
**MPH**	0.342 ± 0.083	╲	0.747	0.809	0.011	1.000	0.685
**GFX+2MDHX**	0.528 ± 0.093	0.747	╲	1.000	1.000	1.000	1.000
**CBG+2MDHX**	0.580 ± 0.091	0.809	1.000	╲	1.000	0.809	1.000
**GFX**	0.654 ± 0.060	**0.011**	1.000	1.000	╲	0.747	1.000
**CBG**	0.293 ± 0.185	1.000	1.000	0.809	0.747	╲	0.689
**2MDHX**	0.577 ± 0.082	0.685	1.000	1.000	1.000	0.689	╲
**Vehicle**	0.231 ± 0.018	0.997	**0.024**	**0.009**	**<0.001**	1.000	**0.006**

Overall Linear Mixed Model result: p<0.001, F_6,106_=15.420. p values of pairwise comparisons are reported in the columns next the mean ± SEM.

**Table 6:** Comparison of treatment effects on spatial WM-related DAR task performance (i.e., alternation rate).

		p-value			
	mean ± SEM	MPH	GFX+2 MDHX	GFX	2MDHX
**MPH**	0.595 ± 0.028	╲	1.000	1.000	1.000
**GFX+2MDHX**	0.569 ± 0.080	1.000	╲	1.000	1.000
**GFX**	0.629 ± 0.044	1.000	1.000	╲	1.000
**2MDHX**	0.553 ± 0.046	1.000	1.000	1.000	╲
**Vehicle**	0.647 ± 0.020	1.000	1.000	1.000	0.542

Overall Linear Mixed Model result: p=0.257, F_4,53_=1.368. p values of pairwise comparisons are reported in the columns next the mean ± SEM.

## Data Availability

All the data supporting the findings of this study are contained within the paper. The raw data will be made available by the authors, without undue reservation.
